# Interobserver and intraobserver reliabilities of determining the ventilatory thresholds in subjects with a lower limb amputation and able-bodied subjects during a peak exercise test on the combined arm-leg (Cruiser) ergometer

**DOI:** 10.1097/MRR.0000000000000536

**Published:** 2022-06-29

**Authors:** Elisabeth K. Simmelink, Pieter U. Dijkstra, Matthijs C. de Bruijn, Jan H.B. Geertzen, Lucas H.V van der Woude, Johan B. Wempe, Rienk Dekker

**Affiliations:** Departments of a Rehabilitation Medicine; b Oral and Maxillofacial Surgery, University Medical Center Groningen, University of Groningen; c Department of Sport Medicine, Martini Sports Medical Center, Martini Hospital; d Center for Human Movement Sciences, University Medical Center Groningen, University of Groningen; e Department of Pulmonary Diseases, University Medical Center Groningen, University of Groningen, Groningen, The Netherlands

**Keywords:** ergometer, exercise test, exercise training, lower limb amputation, ventilatory thresholds

## Abstract

The first (VT1) and second ventilator (VT2) (anaerobic) thresholds are used to individually prescribe exercise training programs. The purpose of this research was to analyze inter- and intraobserver reliabilities of determining VT1 and VT2 in subjects with lower limb amputation (LLA) and able-bodied (AB) subjects during a peak exercise test on the arm-leg (Cruiser) ergometer. Previously published data of exercise tests on the Cruiser ergometer of subjects with LLA (*n* = 17) and AB subjects (*n* = 30) were analyzed twice by two observers. The VT1 and VT2 were determined based on ventilation plots. Differences in determining the VT1 and VT2 between the observers for the first and second analyses were analyzed. To quantify variation in measurement a variance component analysis was performed. Bland–Altmann plots were made, and limits of agreement were calculated. The number of observations in which thresholds could not be determined differed significantly between observers and analysis. Variation in VT1 between and within observers was small (0–1.6%) compared with the total variation, for both the subjects with an LLA and AB subjects. The reliability coefficient for VT1 was more than 0.75, and the limits of agreement were good. In conclusion, based on the results of this study on a population level, VT1 can be used to prescribe exercise training programs after an LLA. In the current study, the determination of VT2 was less reliable than VT1. More research is needed into the clinical application of VT1 and VT2 during a peak exercise test on the Cruiser ergometer.

## Introduction

Most persons with a lower limb amputation (LLA) are elderly with a high prevalence of cardiovascular disease and low physical fitness [1–3]. Low physical fitness results in an undesirable decrease of activities and participation [4,5]. Therefore, it is important for persons with an LLA to start exercising before or as soon as possible after amputation in a safe, comfortable, and efficient manner to improve physical fitness. Before starting exercise training, a valid, reliable and safe physical exercise test especially with regard to cardiovascular risks is required to design exercise training programs. Additionally, outcomes of exercise tests should help to predict successful ambulation with a prosthesis [6–8].

To stimulate physical fitness as soon as possible after surgery, persons with an LLA have to be able to perform large muscle exercises even in the absence of a prosthesis. Testing and exercising on a bicycle ergometer is limited as persons with an LLA cannot make a complete cycling movement with one leg without help, and a limited muscle mass is used because of the amputation [8]. The Cruiser ergometer, a combined arm-leg ergometer, is appropriate for persons with a unilateral LLA to test physical fitness [9–13]. Advantages of the Cruiser ergometer are that persons with an LLA can sit on it with adequate back support and support for the residual limb [13]; they can safely exercise with one leg, both arms and trunk without help of a test assistant and they use relatively large muscle mass.

A protocol for peak cardiopulmonary exercise testing (CPET) can be applied to the Cruiser ergometer [13]. This protocol is used in rehabilitation for the evaluation of exercise intolerance and exercise-related symptoms, which cannot be determined by means of resting pulmonary and cardiac function testing or submaximal testing [14,15]. Furthermore, on basis of such a peak exercise test, the three-phase model of lactic acid accumulation potentially allows the determination of a first ventilatory (VT1) and second ventilatory (VT2) or anaerobic threshold (AT) also for subjects with an LLA. VT1 and VT2 are used especially in cardiac rehabilitation to prescribe exercise training programs individually as it has been described in detail in the extensive review of Binder *et al*. [16]. The prescription of correct exercise intensity is very important during rehabilitation programs. The prescription based on VT1 and VT2 is expected to elicit better responses in the improvement of exercise capacity after training than exercise prescription based on maximal oxygen uptake or maximum heart rate (HR max) [17,18]. The VT1 and VT2 increase with age and are lower in arm exercises compared with leg exercises. Additionally, they are protocol- and disability-specific [14]. It is unknown whether VT1 and VT2 can be reliably determined during peak exercise testing on the Cruiser ergometer. In addition, it is unknown whether differences exist in the level and correct determination of VT1 and VT2 between observers and if there are differences between the subjects with LLA and able-bodied (AB) subjects.

The aims of the study were, therefore, to analyze inter- and intraobserver reliabilities of determining VT1 and VT2 in subjects with an LLA and AB subjects exercising on the Cruiser ergometer by two observers and, second, to analyze differences in VT1 and VT2 between the subjects with an LLA and AB subjects.

## Methods

### Study design

The current study is based on reanalysis of data of two studies regarding standardized peak cardiopulmonary exercise tests on the Cruiser ergometer [10,13]. Inclusion criteria for the subjects with LLA were: age between 18 and 75 years and a unilateral transfemoral amputation, knee disarticulation, or transtibial amputation. Exclusion criteria for the subjects with LLA were: coronary heart disease, clinically relevant arrhythmia, hypertension (DBP > 100 mmHg or SBP > 180 mmHg), pulmonary embolism less than 6 weeks ago, bilateral LLA, upper limb amputation, and cognitive impairments leading to inability to cooperate or inability to obtain consent [13]. The exclusion criteria for the healthy volunteers were age less than 18 years, a body mass index of more than 30, evidence or serious suspicion of cardiovascular diseases, stress or exercise-related pain in the chest, pulmonary diseases, resting blood pressure greater than 140/90, viral or bacterial infection for less than 10 days, use of medication for cardiopulmonary diseases, balance disorders and wounds on the legs and joint diseases [10]. The 17 subjects with an LLA were 14 men and 3 women (Table 1). The cause of the amputation was trauma (11 subjects), cancer (two subjects), vascular reasons (two subjects), pain syndrome (one subject) or neurofibromatosis (one subject) [13]. One subject had a BMI of more than 30 and two of the subjects in which the cause of the amputation was cancer and trauma, respectively, used a β-blocker because of cardiovascular disease [13]. The 30 volunteers were 16 men and 14 women (Table 1). Two observers, a sports physician (>5 year experience) and a rehabilitation physician (1 year experience), determined following a standardized protocol, the VT1 and VT2 of both groups on two occasions at least 3 months apart. The observers independently analyzed data were blinded for the results of each other and for their own results of the first assessment.

**Table 1:** Subject characteristics

	Subjects with LLA (*n* = 17)	AB subjects (*n* = 30)	Significance (*P*)	95% confidence interval of the difference
**Men/women**	14/3	16/14	0.06^a^	
**Mean (SD) age in years**	54.5 (18.6)	37.0 (10.0)	<0.001	9.4–25.7
**Range**	25–80	20–61		
**Mean (SD) BMI in kg/m^2^**	25.2 (4.0)	24.7 (2.5)	0.60	−1.4 to 2.4
**Range**	18.4–31.2	19.3–29.3		
**Median (IQR) time since amputation (months**)	84.0 (4–144)	NA		
**Range**	2–372			

AB, able bodied; IQR, interquartile range; LLA, lower limb amputation; NA, not applicable.

a Based on Fisher exact, all other *P* values based on independent sample *t*-test.

The Medical Ethics Committee (METc) of the UMC Groningen had approved those studies (METc 2011/123 and METc 2005/237) and gave permission to reanalyze the data for this study.

### Instruments and test protocol

The Cruiser ergometer [10,13] (Enraf-Nonius serial number: 3800EN014, Delft, The Netherlands) was used for the peak exercise test in both groups. The test protocol differed somewhat between the groups. Subjects with LLA started with 3 min rest followed by a 3 min warm-up at 20 W. After the warm-up, the workload was increased by 10 W/min until the point of exhaustion was reached or until the physician stopped the test. After completing the test, subjects were observed for another 3 min [13]. For the AB subjects, the test started with 3-min rest, followed by a 5-min warm-up at 50 W. After the warm-up, the workload was increased by 30 W/min for men and 20 W/min for women until the point of exhaustion was reached or until the physician stopped the test. After the exercise test was terminated, a cooling down of 3 min was performed at 20 W [10]. Reasons to terminate testing were inability to maintain 50 revolutions per minute (rpm) (38–53% in subjects with LLA), muscle fatigue in arms (5.9–6.3% in subjects with LLA and 30–37% in AB subjects), muscle fatigue in the leg (17.7–18.8% in subjects with LLA and 13–20% in AB subjects), arm and leg fatigue (37–40% in AB subjects) or severe dyspnea (5.9–6.3% in subjects with LLA and 7–10% in AB subjects) [10,13]. In subjects with LLA, testing was also stopped in 18–31% by the investigator in case of ECG abnormalities, mostly because the ECG was affected by muscle activity of the arms and thorax [13].

The feet of the user were placed against a fixed footrest on the Cruiser ergometer, which can be adjusted to the subjects’ length. The subjects with an LLA performed the test without prosthesis. The residual limb rested on a support. The footrest was used to push off and move the seat backward. The handlebars are used to pull the seat forward again. In this way, arms, trunk and leg(s) overcome resistance provided by the ergometer in a cyclic multilimb movement pattern. The ergometer was set in a constant power mode of between 35 and 60 rpm, and subjects were instructed to maintain a cadence of 50 rpm [10,13]. The accuracy of the Cruiser ergometer is within ±10% power output (W) and ±2 rpm for cadence [19]. Cardiorespiratory outcomes were recorded using an Oxycon Delta (Jaeger, Bunnik, The Netherlands). Subjects wore a face mask and ventilation (VE, in l/min), oxygen uptake (VO_2_, in l/min) and carbon dioxide output (VCO_2_, in l/min) were measured breath by breath and plotted. Peak VO_2_ and peak VCO_2_ were defined as the highest average values obtained over a 30 s period. Blood pressure was measured manually at the beginning of the test, immediately after the test was completed, and after the cooling down period. HR (HR in beats/min) was continuously monitored using a 12-lead ECG [10,13].

### Determination of the ventilatory (anaerobic) thresholds

The VT1 and VT2 were determined based on ventilation plots as described by Wasserman *et al*. [20]. The VT1 was determined using three criteria, (a) intersection of a two line regression of the VCO_2_ versus VO_2_ (V-slope) graph, with a change of the slope from less than one to equal to one or greater than one, (b) first increase of VE/VO_2_ versus workload (W) without a simultaneous increase in VE/VCO_2_, and (c) first rise of fraction of oxygen in the expired air (PETO2), whereas the fraction of CO2 in the expired air (PETCO2) remains constant or is increasing. The VT2 was determined using three criteria, (a) inflection of VE versus VCO_2_ (VE/VCO_2_ slope), (b) nonlinear increase of VE/VCO_2_ versus W and (c) deflection point of the end-tidal PETCO_2_ [16]. The criteria are in order of preference: when the first criterion yielded adequately positioning, it was chosen; when the first criterion could not be reasonably applied, the second criterion was chosen, and so further. The observers assessed all three plots for each threshold and based their decision on the V-slope or the ventilatory equivalents; depending on which plot most clearly showed that particular VT1 or VT2. For each VT1 or VT2 determined, the Oxycon Delta software calculated the VO_2_ and HR. If an observer could not determine VT1 or VT2, it was recorded.

### Statistical analysis

Differences between subjects with an LLA and AB, subjects were analyzed by means of *t*-test for independent samples and Chi-square tests. Differences in determining the VT1 and VT2 between the observers for the first and second analyses were analyzed using Cochran’s Q test with a post hoc pairwise with Bonferroni correction. To quantify variation in measurement results, a variance component analysis (restricted maximum likelihood method) was performed for subjects with an LLA and AB subjects separately. Sources of variation included subjects (persons differ from each other) and observers (observers determine thresholds differently) and repeated analysis (results of the first vs. second analysis). Negative variance components were set to 0. Based on the results of the variance components, the error variance was calculated as the sum of the variances minus the variance due to subjects with LLA. The error variance was, thereafter, divided by the sum of variances, resulting in a reliability coefficient. The following interpretation was used for the reliability coefficient with minimum reliability of 0.7 and clinically relevant at 0.9 [21]. For AB subjects, a similar procedure was followed. Limits of agreement were calculated, and Bland–Altman plots were drawn for the outcomes percentage of VO_2_ and HR on the VT1 and VT2. Statistical analyses were performed using SPSS (IBM SPSS Statistics 23, New York, USA).

## Results

Baseline data of the subjects are presented in Table 1. Subjects with an LLA were significantly older than the AB subjects.

The number of observations in which thresholds could not be determined differed significantly between observers and tests (Cochran’s Q test, *P* < 0.001). The number of observations in which the thresholds could not be determined was higher for VT2 than that for VT1. The number of observations in which thresholds could not be determined was significantly lower for observer 1 compared with observer 2 in analysis 1 (*P* = 0.008) and analysis 2 (*P* = 0.001) (Table 2).

**Table 2:** Number of observations in which observers could not determine the first ventilatory threshold (VT1) and/or the second ventilatory threshold (VT2) for all subjects (*n* = 47)

Observer 1			Observer 2	
	Analysis 1	Analysis 2	Analysis 1	Analysis 2
VT1	2	2	3	4
VT2	6^a^	4^b^	16^a^	12^b^

a Difference between observer 1 and 2 is significant (*P* = 0.008).

b Difference between observer 1 and 2 is significant (*P* = 0.001) Based on Cochran’s Q test (*P* < 0.001) and post hoc pair wise comparison with a Bonferroni correction for pairwise comparison.

### Inter- and intraobserver reliabilities

Variation between and within observers was small (0–1.6%) compared with the total variation, for both subjects with an LLA and AB subjects (Table 3). The reliability coefficients for VT1, expressed as VO_2_ and HR at that threshold were, respectively, 0.89 and 0.75 for the subjects with an LLA and 0.81 and 0.84 for the AB subjects. The VT1 expressed as VO2 and HR variation between and within observers are described in Table 3. Variance components for VT2 were also calculated (Table 3) but were based on a limited number of observations.

**Table 3:** Variance component analysis (Restricted Maximum Likelihood Method) for subjects with lower limb amputation (LLA) and able-bodied subjects (AB)

Sources of variation	LLA	LLA	LLA	AB	AB	AB
		% of total var	% of error var		% of total var	% of error var
VT1 VO_2_ (l/min)						
Subject	114.42	88.6		192.38	81.1	
Observer	0.0	0.0	0.0	1.77	0.7	3.9
Analysis	0.97	0.7	6.6	0.0	0.0	0.0
Observer × subject	5.58	4.3	37.9	0.0	0.0	0.0
Subject × analysis	0.0	0.0	0.0	21.08	8.9	46.9
Observer × analysis	0.20	0.2	1.4	0.0	0.0	0.0
Residual	7.95	6.2	54.1	22.11	9.3	49.2
Sum of var. comp.	129.12			237.34		
Reliability coeff.	0.89			0.81		
VT1 HR (beats/min)						
Subject	263.6	74.6		221.1	84.0	
Observer	1.1	0.3	1.2	4.3	1.6	10.1
Analysis	0.0	0.0	0.0	0.0	0.0	0.0
Observer × subject	27.7	7.8	30.8	9.4	3.6	22.3
Subject × analysis	6.7	1.9	7.4	7.1	2.7	16.8
Observer × analysis	0.0	0.0	0.0	0.0	0.0	0.0
Residual	54.4	15.4	60.6	21.5	8.2	50.8
Sum of var. comp.	353.4			265.4		
Reliability coeff.	0.75			0.84		
VT2 VO_2_ (ml/min)						
Subject	258.11	99.4		327.59	93.7	
Observer	0.0	0.0	0.0	0.0	0.0	0.0
Analysis	0.0	0.0	0.0	0.0	0.0	0.0
Observer × subject	0.0	0.0	0.0	4.96	1.4	22.3
Subject × analysis	0.04	0.0	2.4	5.54	1.6	25.0
Observer × analysis	0.0	0.0	0.0	0.0	0.0	0.0
Residual	1.49	0.6	97.6	11.71	3.3	52.7
Sum of var. comp.	259.64			349.80		
Reliability coeff.	0.99			0.94		
VT2 HR (beats/min)						
Subject	1028.6	99.6		181.7	82.3	
Observer	0.0	0.0	0.0	0.0	0.0	0.0
Analysis	0.0	0.0	0.0	1.3	0.6	3.4
Observer × subject	0.0	0.0	0.0	6.4	2.9	16.3
Subject × analysis	0.0	0.0	0.0	13.9	6.3	35.6
Observer × analysis	0.0	0.0	0.0	0.25	0.1	0.6
Residual	4.1	0.4	100.0	17.2	7.8	44.0
Sum of var. comp.	1032.7			220.8		
Reliability coeff.	1.00			0.82		

coeff., coefficient; HR, heart rate; sum of var. comp, sum of variance component analysis; VO_2_, oxygen uptake; VT1, first ventilatory threshold; VT2, secondary ventilatory threshold.

**Fig. 1:**
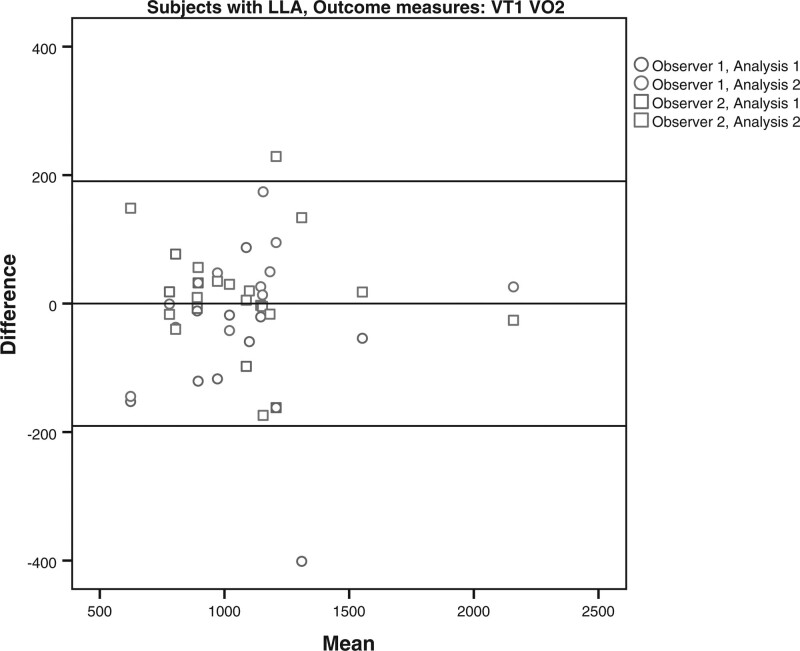
Bland and Altman plots for oxygen uptake (VO2) at the the first ventilatory threshold (VT1) during the test on the Cruiser ergometer for subjects with a lower limb amputation.

**Fig. 2:**
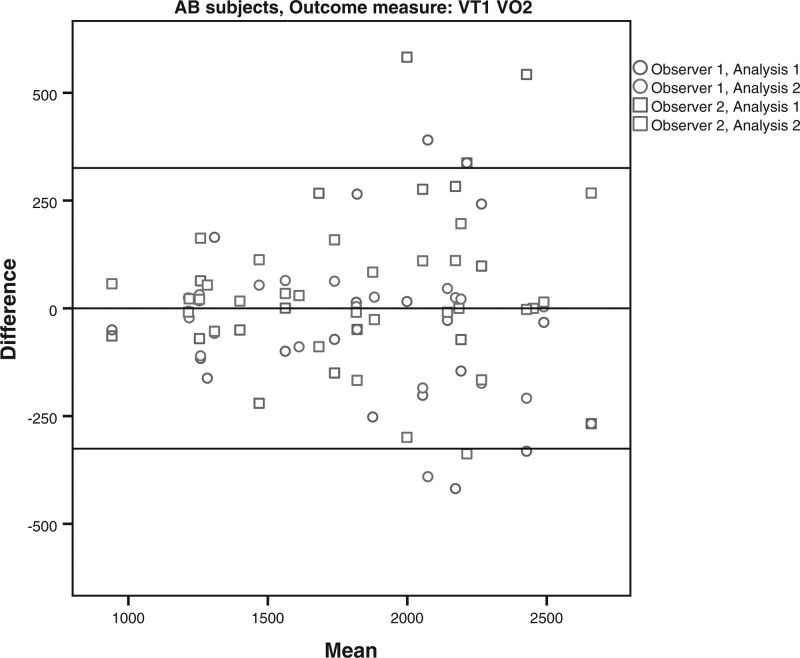
Bland and Altman plots for oxygen uptake (VO2) at the the first ventilatory threshold (VT1) during the test on the Cruiser ergometer for the able-bodied subjects.

**Fig. 3:**
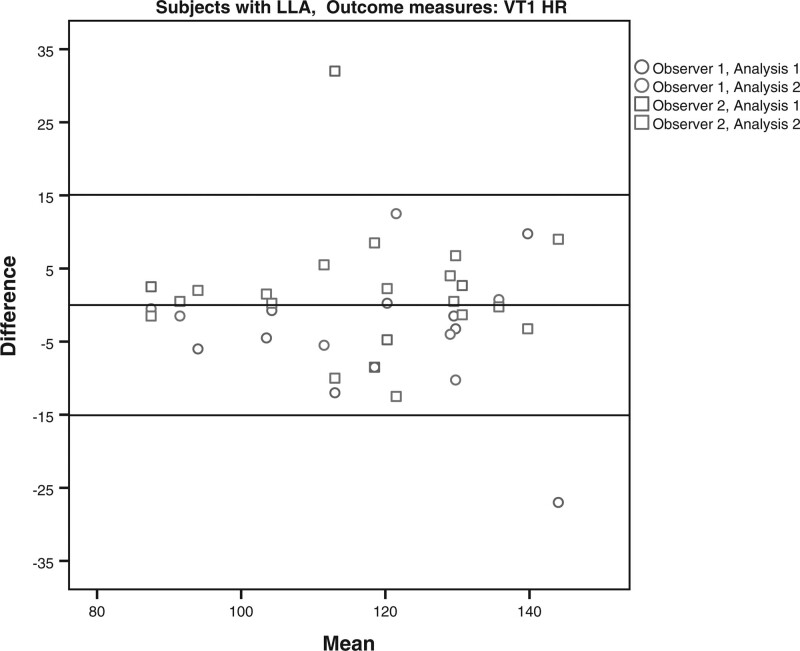
Bland and Altman plots for heart rate(HR) at the first ventilatory threshold (VT1) during the test on the Cruiser ergometer for subjects with a lower limb amputation.

**Fig. 4:**
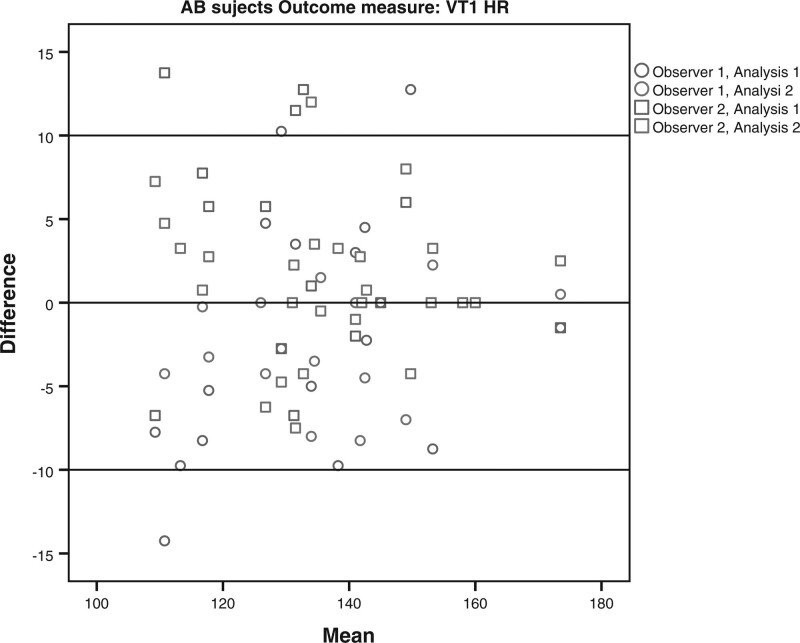
Bland and Altman plots for heart rate(HR) at the first ventilatory threshold (VT1) during the test on the Cruiser ergometer for able-bodied subjects.

**Fig. 5:**
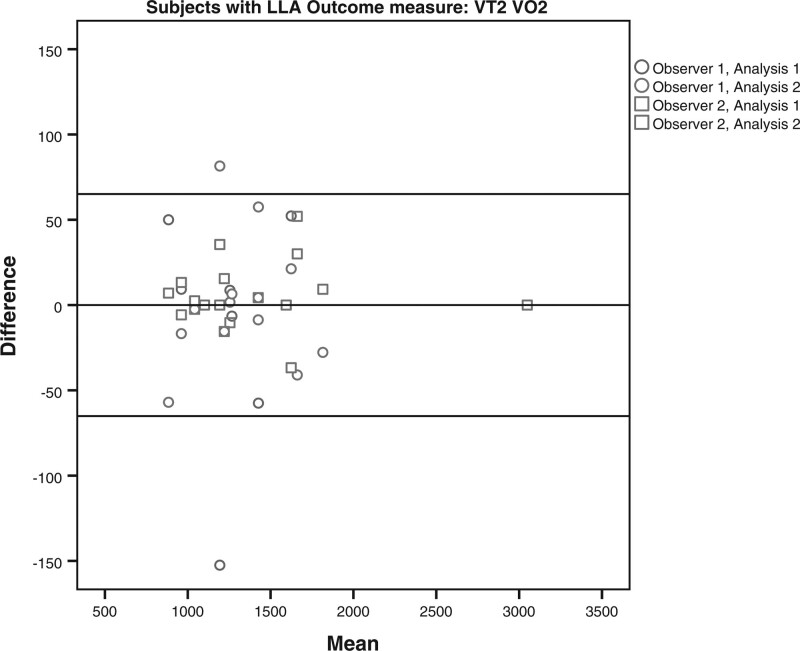
Bland and Altman plots for oxygen uptake (VO2) at the second ventilatory threshold (VT2) on the Cruiser ergometer for subjects with a lower limb amputation.

**Fig. 6:**
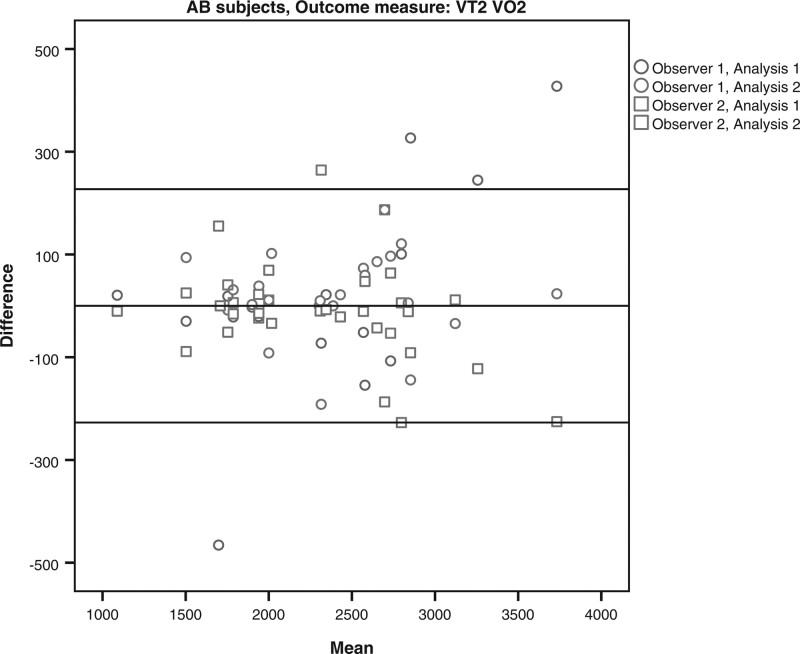
Bland and Altman plots for oxygen uptake (VO2) at the second ventilatory threshold (VT2) on the Cruiser ergometer for the able-bodied subjects.

**Fig. 7:**
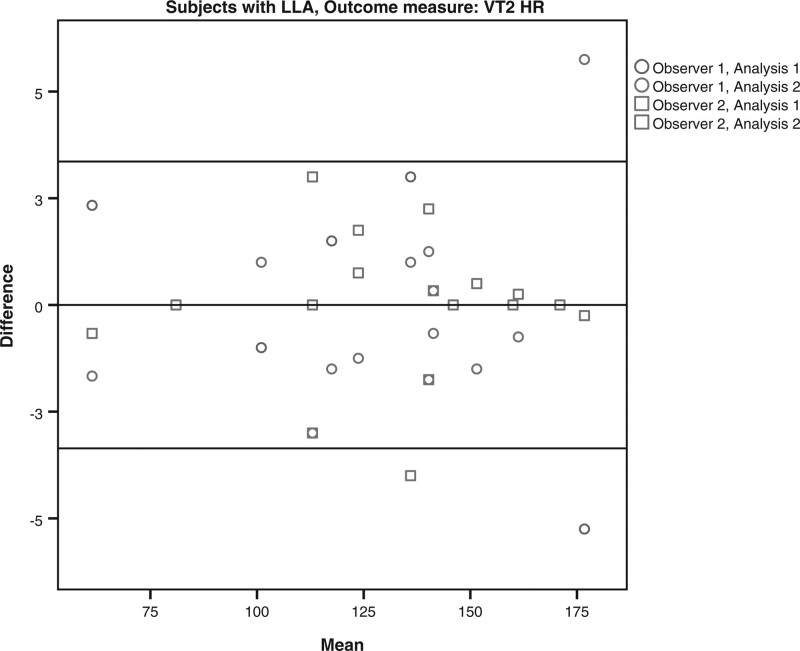
Bland and Altman plots for heart rate (HR) at the second ventilatory threshold (VT2) on the Cruiser ergometer for subjects with a lower limb amputation.

**Fig. 8:**
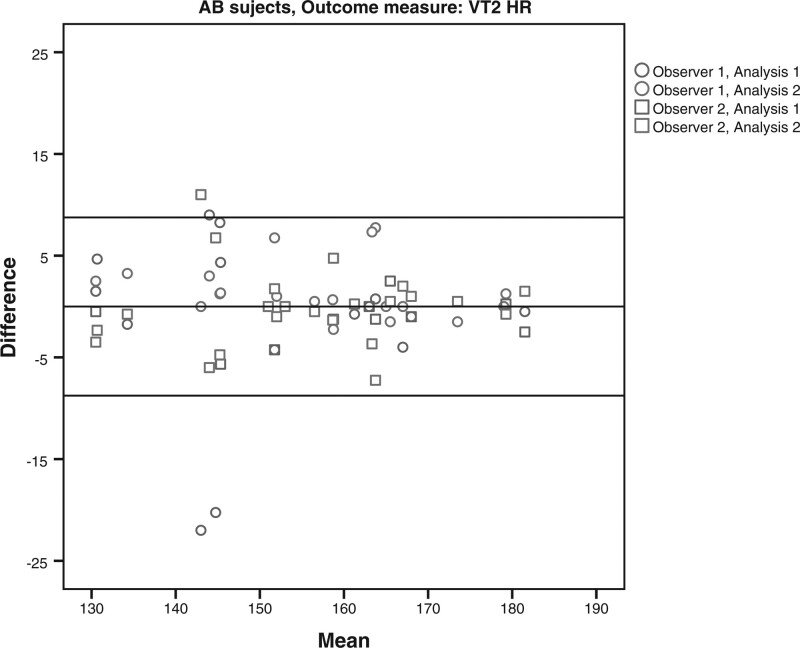
Bland and Altman plots for heart rate (HR) at the second ventilatory threshold (VT2) on the Cruiser ergometer for the able-bodied subjects.

The limits of agreement for the VO2 and HR at VT1 and VT2 for the subjects with LLA and AB subjects are shown in Table 4 and Figs. 1–8. There were varying differences in the limits of agreements between the subjects with an LLA and the AB subjects (Table 4). In the plots, there were no obvious differences between the observers (interobserver reliability) and between the analysis (intraobserver reliability) (Figs. 1–8).

**Table 4:** Limits of agreement of the Bland and Altman plots

Limits of agreement (±1.96 SD)	LLA subjects	AB subjects
VT1 VO_2_ (l/min)	0.19	0.33
VT1 HR (beats/min)	15.08	10.04
VT2 VO_2_ (l/min)	0.07	0.23
VT2 HR (beats/min)	3.36	9.19

AB, able bodied; HR, heart rate; LLA, lower limb amputation; VO_2_, oxygen uptake; VT1, first ventilatory threshold; VT2, second ventilatory threshold.

All the outcome parameters at VT1 and VT2 were significantly higher for the AB subjects than for subjects with LLA (Table 5).

**Table 5:** Differences of the outcome parameters between able-bodied subjects and subjects with lower limb amputation

	Subjects with LLA (*n* = 17)	AB subjects (*n* = 30)	Significance (*P*)	95 % confidence interval of the difference
	Mean (SD)	Mean (SD)		
**VT1 VO_2_ (l/min**)	1.12 (0.35)	1.82 (0.46)	<0.001	0.45–0.96
**VT1HR (beats/min**)	117.9 (17.2)	136.9.1 (15.3)	<0.001	9.2–28.9
**VT2 VO**_**2**_ **(l/min**)	1.42 (0.51)	2.33 (0.58)	<0.001	0.56–1.26
**VT2HR (beats/min**)	130.9 (32.1)	156.2 (14.1)	0.001	11.2–39.3

AB, able bodied; HR, heart rate; LLA, lower limb amputation; VO_2_, oxygen uptake; VT1, first ventilatory threshold; VT2, second ventilatory threshold.

## Discussion

Reliability coefficients of VT1 in this study were all above the minimally required 0.7, but also below 0.9. The latter is generally viewed as a requirement for clinical application [21]. On a population level, VT1 can be used to prescribe exercise training programs, but based on the current study, the VT1 is not applicable in individual cases. The values of VO2 and HR at VT1 and VT2 were higher for the AB subjects than for the subjects with LLA. VT1 could be determined more reliably than VT2. There was a difference between the number of observations in which observers could determine VT2: observer 1 could determine VT2 in 87.2 and 91.5% (first and second analyses) and observer 2 in 66.0 and 74.5% (first and second analyses). Because VT2 could not be determined in all subjects, the number of observations involved in the variance components analysis and Bland–Altman analysis was considerably smaller (Table 2), and the analysis of the inter- and intraobserver reliabilities of the VT2 was limited.

In a similar study determining ventilatory thresholds in individuals with spinal cord injury, about 90% of the ventilatory thresholds could be determined, particularly the VT2 in individuals with tetraplegia could not be determined [22]. In that study, intraobserver reliability for determining ventilatory thresholds was good, as in our study for the VT1. In a study into the efficacy of the one-leg cycling test for determining the AT of LLA, the correlation coefficient between the first and second AT values was 0.962. However, in that study only AT, which is the same as VT1, was determined and not VT2 [23].

In the current study, the VO_2_ at VT1 and VT2 was higher for the AB subjects in comparison with the subjects with an LLA. In addition to age, probably the LLA had not only an effect on VO_2_ peak [13], but also on ventilatory thresholds. Subjects with LLA performed the exercise test on the Cruiser ergometer with one leg and two arms and the AB subjects with two legs and two arms. During upper body exercise, ventilatory threshold can be reached at lower absolute VO_2_ than during lower body exercise, whereas VT1 and VT2 occur at similar percentage of VO_2_ max for both modes of exercise performed [24]. Subjects with LLA use one leg and they had to exercise relatively more with their upper body. This difference in upper body exercise may explain lower values of VO_2_ at VT1 and VT2 for subjects with LLA compared with AB subjects.

### Limitations

A study limitation is the age difference between subjects with LLA and AB subjects. As mentioned above, it was expected that the VO_2_ at VT1 and VT2 was higher for the AB subjects than for subjects with LLA because of the difference in performing the exercise test with one or two legs. However, this age difference can also explain the difference in VO_2_. Furthermore, the sample size of persons with an LLA was small, and especially for the determining of inter- and intraobserver reliabilities of the VT2, the calculations were limited due to missing data (not being able to determine VT2). The main reason for amputation in our study population was traumatic. In contrast, the main reason for amputation in the general population of persons with LLA is vascular. However, we think our sample of younger LLA subjects is representative for the subjects with an LLA who participate in a rehabilitation program to regain walking functionality because many older subjects with a vascular LLA die within a year after the amputation and/or do not receive rehabilitation after amputation [25–27]. Another limitation of this study was that data of performing CPET on the Cruiser ergometer twice in the same circumstances were not analyzed, so test–retest reliabilities of determining the VT1 and VT2 were not investigated. In addition, the outcome measure power output or work rate expressed in Watt was not used as an outcome measure because the software of the Oxycon Delta was not linked to the Cruiser ergometer and, consequently, the power output was not automatically described on VT1 and VT2. This shortcoming is a limitation because power output can be important as an outcome measure in exercise intensity prescription, especially if the HR is influenced by medication [20]. Furthermore, a limitation was the difference in test protocol between the subjects with an LLA and AB subjects. All the subjects with LLA had the same test protocol, and for AB subjects, there was a difference in test protocol for men and women.

### Future research

For exercise prescription, it is mostly recommended to use the VT1 and VT2 [16]. Based on the results of this study for the Cruiser ergometer, the use of only the VT1 is recommended, but further research on the clinical application is needed because the reliability coefficients were not above 0.9. To use the VT2, more research is needed to establish the reliability of determining VT2 in a larger sample of subjects with LLA performing a CPET on the cruiser ergometer. In this study, it was not clear if VT2 was not reached because the subjects did not reach the threshold or the observer could not determine the threshold. Based on a CPET at the start of the rehabilitation after an LLA, an individualized exercise intensity program can be composed to improve physical fitness, as an important cornerstone of recovery. In the present study, it was not investigated whether such an exercise intensity prescription based on VT1 or VT2 is more favorable to exercise prescription based on other variables such as rate of perceived exertion, percentage of HR reserve or percentage of peak power output. This difference should be investigated in future research. Also, training regimens based on VT1 and VT2, resulting from CPET on a Cruiser ergometer, should be clinically evaluated on individual level.

### Conclusion

Based on the intra- and interobserver reliabilities determined in this study, VT1 can be used to prescribe exercise training programs after an LLA on population level. In the current study, determination of VT2 was less reliable than VT1. More research is needed into the clinical application of VT1 and VT2 during a peak exercise test on the Cruiser ergometer.

## Acknowledgements

### Conflicts of interest

There are no conflicts of interest.
